# Significance of D-Dimer in Acute Ischemic Stroke Patients With Large Vessel Occlusion Accompanied by Active Cancer

**DOI:** 10.3389/fneur.2022.843871

**Published:** 2022-03-23

**Authors:** Kwang Hyun Pan, Jaeyoun Kim, Jong-Won Chung, Keon Ha Kim, Oh Young Bang, Pyoung Jeon, Gyeong-Moon Kim, Woo-Keun Seo

**Affiliations:** ^1^Department of Neurology, Samsung Medical Center, Sungkyunkwan University School of Medicine, Seoul, South Korea; ^2^Department of Neurology, Anam Hospital, Korea University, Seoul, South Korea; ^3^Department of Radiology, Stroke and Cerebrovascular Center, Samsung Medical Center, Sungkyunkwan University School of Medicine, Seoul, South Korea

**Keywords:** cancer-related stroke, D-dimer, endovascular thrombectomy, large vessel occlusion, recanalization

## Abstract

**Background:**

This study aimed to investigate clinical outcome predictors of acute stroke patients with large vessel occlusion and active cancer and validate the significance of D-dimer levels for endovascular thrombectomy decisions.

**Methods:**

We analyzed a prospectively collected hospital-based stroke registry to determine clinical EVT outcomes of acute stroke patients within 24 h with following criteria: age ≥18 years, NIHSS ≥6, and internal carotid artery or middle cerebral artery lesion. All patients were classified into EVT and non-EVT groups. Patients were divided into two groups by initial D-dimer level. We explored variables potentially associated with successful recanalization as well as 3-month functional outcomes and mortality rates.

**Results:**

Among 68 patients, 36 were treated with EVT, with successful recanalization in 55.6%. The low D-dimer group showed a higher rate of successful recanalization and favorable outcome than the high D-dimer group. The mortality rate was higher in the high D-dimer group. No EVT and high D-dimer level were independent predictors of mortality, whereas lesion volume and low D-dimer level were independently associated with favorable outcomes.

**Conclusions:**

D-dimer level is a prognostic factor in acute LVO stroke patients with active cancer, and its high value for EVT decisions provisionally supports its testing in this patient population.

## Introduction

Endovascular thrombectomy (EVT) is an established treatment modality for acute stroke patients with large vessel occlusion (LVO) (1). In acute stroke with LVO, current guidelines recommend considering EVT as positively as possible if it meets the criteria. However, there are many unanswered questions regarding the choice of EVT in acute stroke patients with active cancer.

There has been a growing number of acute ischemic stroke patients with active cancer due to advances in cancer treatment and the expanding aging population. About 5% of hospitalized ischemic stroke patients have comorbid cancer; among them, 3.1% have LVO treated by EVT (2). Ischemic stroke associated with active cancer has different behaviors due to the specific etiologic mechanism and outcomes. Stroke patients with active cancer usually have poorer outcomes, have high mortality rates, and are at a higher risk of bleeding complications than non-cancer stroke patients (3). In the choice of acute or chronic therapy for stroke patients with active cancer, cancer-related coagulopathy and anti-cancer treatments such as chemotherapy and radiotherapy should be considered (4). In addition, the short life expectancy of advanced cancer patients makes it difficult to choose aggressive therapy, such as intravenous thrombolysis and endovascular intervention, in the setting of an acute stroke. Therefore, the paucity of evidence makes it difficult to determine EVT in acute stroke patients with active cancer. Previous studies stressed the need for cautious patient selection for EVT among those with active cancer because of poor prognosis despite the similar recanalization rate of cancer patients (5, 6).

We, thus, aimed to explore the overall clinical outcomes and predictors of LVO in patients with acute stroke and active cancer. In particular, D-dimer level is a well-known prognosticator in stroke patients with active cancer (7, 8). Therefore, this study focused on the clinical implication of baseline D-dimer level on the choice of EVT among acute stroke patients with active cancer.

## Methods

### Study Design and Patient Selection

This was retrospective analysis of a prospectively collected stroke registry based on a single tertiary hospital that enrolled patients with acute stroke or transient ischemic attack within 7 days from February 2011 to April 2020. The institutional review board of Samsung Medical Center approved the study protocol (2016-08-064). Because anonymized and open data were used in this study, the board waived the requirement for informed consent. All research was conducted in accordance with the principles of the Declaration of Helsinki. In this study, we screened the participants according to the following criteria: age ≥18 years; NIHSS score ≥6; lesion vessel in the internal carotid artery, middle cerebral artery M1 or M2 segment, time of stroke onset within 24 h, and presence of active cancer at presentation. We did not limit specific type of cancer. Active cancer was defined when they met the following criteria: newly diagnosed cancer in 6 months; treatment with anti-cancer therapy such as surgery, chemotherapy, and radiation therapy in 6 months; recurrent cancer in 6 months; or metastatic cancer in 6 months. All endovascular procedures were performed by neurointerventionalists discretion. A balloon guide catheter was routinely used, and stent retrievers or contact aspiration with intermediate catheter or combined strategy were used.

### Clinical Outcome Assessment

The dataset obtained from a prospectively collected stroke registry in Samsung Medical Center includes mTICI grade and mRS. We assessed the successful recanalization rate defined as mTICI grade 2b or 3 (9). Hemorrhagic transformation was assessed on computed tomography or magnetic resonance imaging follow-up after EVT. We defined hemorrhagic transformation using ECASS III classification (10). We assessed the NIHSS score at the initial visit and at discharge to analyze short-term neurologic improvement. Clinical outcome measurements at 3 months included assessments of mortality and disability according to the mRS score. We defined an mRS score of 0–2 as a favorable clinical outcome and an mRS score of 3–6 as a poor clinical outcome. We also investigated the cause of death by stroke-related deaths and other medical–related deaths. We sorted deaths directly related to acute stroke such as brain edema or central respiratory failure as stroke-related deaths, and other medical causes related to disease progression of cancer itself as medical–related deaths. Marked neurological improvement was defined as a decrease of 10 points or more in the NIHSS score during the hospital stay or a fully improved neurological deficit.

### Imaging and Laboratory Data

The measurement of the infarct volume was performed using in-house software. The details of the methods and the validity/performance of the software were published elsewhere (11). In brief, the software is developed based on the deep-learning model and can extract the mask images of infarct lesions and the volume of infarction from the diffusion-weighted images.

Laboratory data, such as complete blood counts, D-dimer level, lipid panel, and C-reactive protein level, were obtained from the first blood sample collected at the emergency department.

### Statistical Analysis

Because the primary purpose of this study was to describe the outcomes of acute stroke with LVO and active cancer associated with EVT, patients were classified into EVT and non-EVT groups. We also divided the patients into two groups based on their initial D-dimer levels. The optimal D-dimer cut-off point predicting a 3-month favorable outcome was determined using Youden's J statistics. (sensitivity + specificity:1). Because the optimal cut-off point predicting a 3-month favorable outcome was 3.825 μg/mL, we simply divided the patients into a low initial D-dimer level group (*n* = 25; D-dimer level <4) and a high D-dimer level group (D-dimer level >4). Intergroup comparisons were performed using the Wilcoxon rank-sum test for continuous variables and the χ^2^ test for categorical variables. Univariate and multivariate logistic regression analyses were performed to identify factors associated with poor clinical outcomes. The stepwise forward selection method was used to select variables for the multivariate analysis. The *p*-value threshold for entry was set to 0.05. Propensity score matching was conducted to adjust for age, sex, hypertension, diabetes mellitus, dyslipidemia, and atrial fibrillation between patients treated with EVT and those treated with low and high D-dimer levels. All statistical analyses were performed using SPSS statistics 25.0, and values of *p* < 0.05 were considered statistically significant.

## Results

### Baseline Characteristics

**Figure 1:**
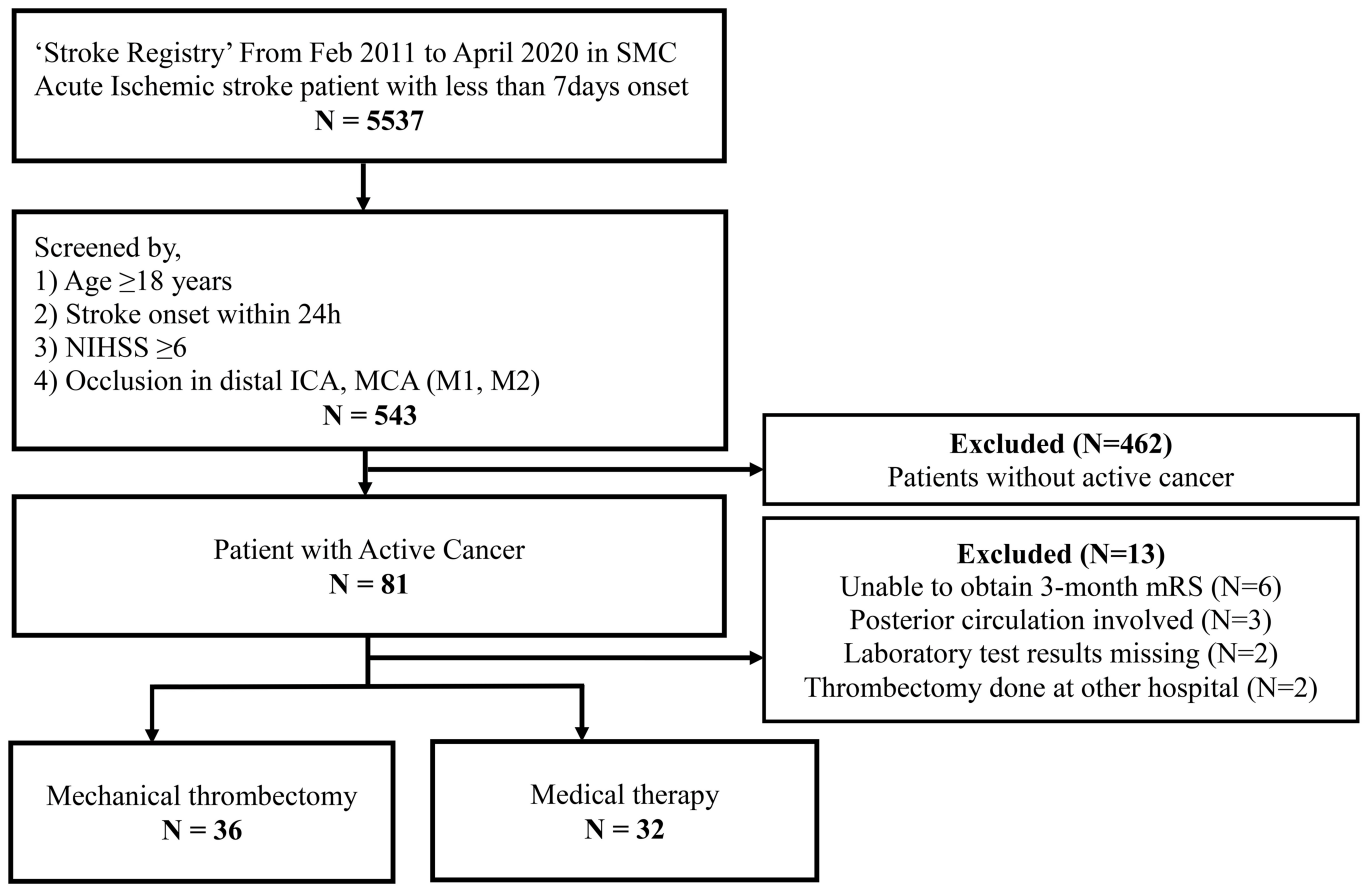
Flow of patient inclusion.

During the study period, a total of 5,537 patients were enrolled in the SMC stroke registry (Figure 1). Among them, 543 patients met the criteria for acute stroke with LVO and 81 patients with active cancer were selected. Finally, we analyzed the data of 68 patients after the exclusion of an additional 13 patients due to no 3-month outcome (*n* = 6), posterior circulation involvement only (*n* = 3), missing laboratory test (*n* = 2), and EVT performed at another hospital (*n* = 2).

The mean patient age was 65.59 years, 36 (52.9%) were male, 32 (47%) had lung cancer, 36 (52.9%) had distant metastasis, and the median initial NIHSS score was 15 (10–20). Thirty-six patients (52.9%) were treated with EVT, while 32 patients (47.1%) were treated medically. The mean initial D-dimer level was 16.07 μg/mL. The baseline characteristics of each group are presented in Table 1. The EVT group had more hypertension and atrial fibrillation, a higher mean platelet count, lower mean low-density lipoprotein (LDL) cholesterol, and lower D-dimer levels than the non-EVT group. The lesion volume was smaller and the onset-to-emergency room time was shorter than that in the non-EVT group. Age, sex, baseline NIHSS score, other comorbid disease, laboratory results, and occluded vessel site did not differ significantly between groups.

**Table 1:** Patients' baseline characteristics.

	**Non-EVT (*n* = 32)**	**EVT (*n* = 36)**	***P*-value**	**D-dimer level <4 (*n* = 25)**	**D-dimer level >4 (*n* = 43)**	***P*-value**
Age, years	63.4 ± 8.5	67.6 ± 10.4	0.08	70.6 ± 10.3	62.67 ± 8.2	0.20
Male sex	16 (50)	20 (55.6)	0.65	12 (48.0)	24 (55.8)	0.53
Baseline NIHSS score	16 (8–20)	14 (10–20)	0.94	13 (11–19)	16 (10–21)	0.88
Hypertension	7 (21.9)	18 (50.0)	0.02	12 (48.0)	13 (30.2)	0.14
Diabetes	5 (15.6)	4 (11.1)	0.58	4 (16.0)	5 (11.6)	0.61
Hyperlipidemia	3 (9.4)	9 (25.0)	0.09	6 (24.0)	6 (14.0)	0.30
Atrial fibrillation	0 (0)	11 (30.6)	<0.01	8 (32.0)	3 (7.0)	<0.01
Previous history of stroke	5 (15.6)	6 (16.7)	0.91	4 (16.0)	7 (16.3)	0.98
Smoking	8 (25.0)	15 (41.7)	0.15	9 (36.0)	14 (32.6)	0.77
Alcohol	3 (9.4)	7 (19.4)	0.24	2 (8.0)	8 (18.6)	0.23
Lung cancer	14 (43.8)	18 (50.0)	0.61	13 (52.0)	19 (44.2)	0.53
Distant metastasis	18 (56.3)	18 (50.0)	0.61	9 (36.0)	27 (62.8)	0.03
Hemoglobin (g/dL)	10.8 ± 2.2	11.4 ± 2.2	0.21	11.95 ± 1.88	10.6 ± 2.3	0.02
Platelets (10^3^/mm^3^)	164.8 ± 125.3	183.2 ± 73.4	0.04	229.48 ± 93.04	142.5 ± 91.8	<0.01
INR	1.3 ± 0.3	1.1 ± 0.2	<0.01	1.05 ± 0.10	1.3 ± 0.3	<0.01
D-dimer (μg/mL)	21.4 ± 18.8	11.3 ± 15.8	0.02	–	–	
LDL cholesterol (mg/dL)	96.5 ± 33.4	81.0 ± 27.8	0.03	90.04 ± 27.33	87.3 ± 33.7	0.34
C-reactive protein (mg/dL)	5.4 ± 5.2	7.1 ± 10.9	0.26	3.73 ± 5.72	7.8 ± 9.7	<0.01
**Occlusion site**						
ICA	5 (15.6)	13 (36.1)	0.06	7 (28.00)	11 (25.6)	0.83
M1	12 (37.5)	15 (41.7)	0.73	10 (40.00)	17 (39.5)	0.97
M2	15 (46.9)	8 (22.2)	0.03	8 (32.00)	15 (34.9)	0.81
Lesion volume (mL)	71.4 ± 63.7	26.0 ± 33.9	<0.01	20.21 ± 22.49	63.1 ± 61.6	<0.01
Onset-to-ER (min)	135 (79–385)	60 (25–113)	<0.01	56 (27–95)	120 (60–335)	0.01
Door-to-puncture (min)	–	119 (97–143)	–	106 (93–122)	134 (100–195)	0.19

*Values are presented as n (%), mean ± standard deviation, or median (interquartile range)*.

*EVT, endovascular therapy; INR, international normalized ratio; LDL, low-density lipoprotein; NIHSS, National Institute of Health Stroke Scale*.

The high D-dimer group had less atrial fibrillation, more distant metastasis, lower mean hemoglobin level, lower mean platelet count, prolonged prothrombin time, and higher mean C-reactive protein levels than the low D-dimer group. The lesion volume was significantly larger and the onset-to-emergency room time was longer than that in the low D-dimer group. Age, sex, baseline NIHSS, other comorbid disease, laboratory results, and occluded vessel site did not differ significantly between the groups.

### Clinical Outcomes

The clinical outcomes of each group are presented in Table 2. Among the 36 patients treated with EVT, 20 (55.6%) had successful recanalization. The low D-dimer group showed a higher successful recanalization rate than the high D-dimer group (73.7 vs. 35.3%, *p* = 0.02). The incidence of hemorrhagic transformation was not significantly different. The EVT group showed a lower mean NIHSS score at discharge than the non-EVT group (median, 6 [interquartile range (IQR), 2–14] vs. 16 [IQR, 7–42], *p* < 0.01), and the high D-dimer group showed a higher mean NIHSS score at discharge than the low D-dimer group (median, 16 [IQR, 7–40] vs. 4 [IQR, 1–13], *p* < 0.001).

**Table 2:** Clinical outcomes by EVT and D-dimer groups.

	**Non-EVT (*n* = 32)**	**EVT (*n* = 36)**	***P*-value**	**D-dimer level <4 (*n* = 25)**	**D-dimer level >4 (*n* = 43)**	***P*-value**
Successful recanalization (mTICI 2b−3)	–	20 (55.6)	–	14 (73.7)*	6 (35.3)*	0.02
Hemorrhagic transformations	5 (15.6)	6 (16.7)	0.91	4 (16.0)	7 (16.3)	0.98
NIHSS at discharge	16 (7–42)	6 (2–14)	<0.01	4 (1– 13)	16 (16–42)	<0.01
Median mRS score at 3 months	6 (5–6)	3.5 (1–6)	<0.01	1 (1–3)	6 (5–6)	<0.01
Favorable outcomes (mRS score of 0–2)	5 (15.6)	13 (36.1)	0.06	15 (60.0)	3 (7.0)	<0.01
Mortality at 3 months	22 (68.8)	12 (33.3)	<0.01	3 (12.0)	31 (72.1)	<0.01

*Values are number (%) or median (interquartile range)*.

*Number of successful recanalization was calculated among EVT group only.

The median 3-month mRS score was significantly better in the EVT group than in the non-EVT group (median, 3.5 [IQR, 1–6] vs. 6 [IQR, 5–6], *p* = 0.01) and significantly better in the low D-dimer group than in the high D-dimer group (median, 1 [IQR, 1–3] vs. 6 [IQR, 5–6], *p* < 0.01). The EVT group had more favorable outcomes, but the difference was not statistically significant. The low D-dimer group had significantly more favorable outcomes than the high D-dimer group. The 3-month mortality rate was significantly higher in the non-EVT group than the EVT group and the high D-dimer group than the low D-dimer group (*p* < 0.01) (68.8 vs. 33.3%, 58.9 vs. 10.5%, respectively).

**Figure 2:**
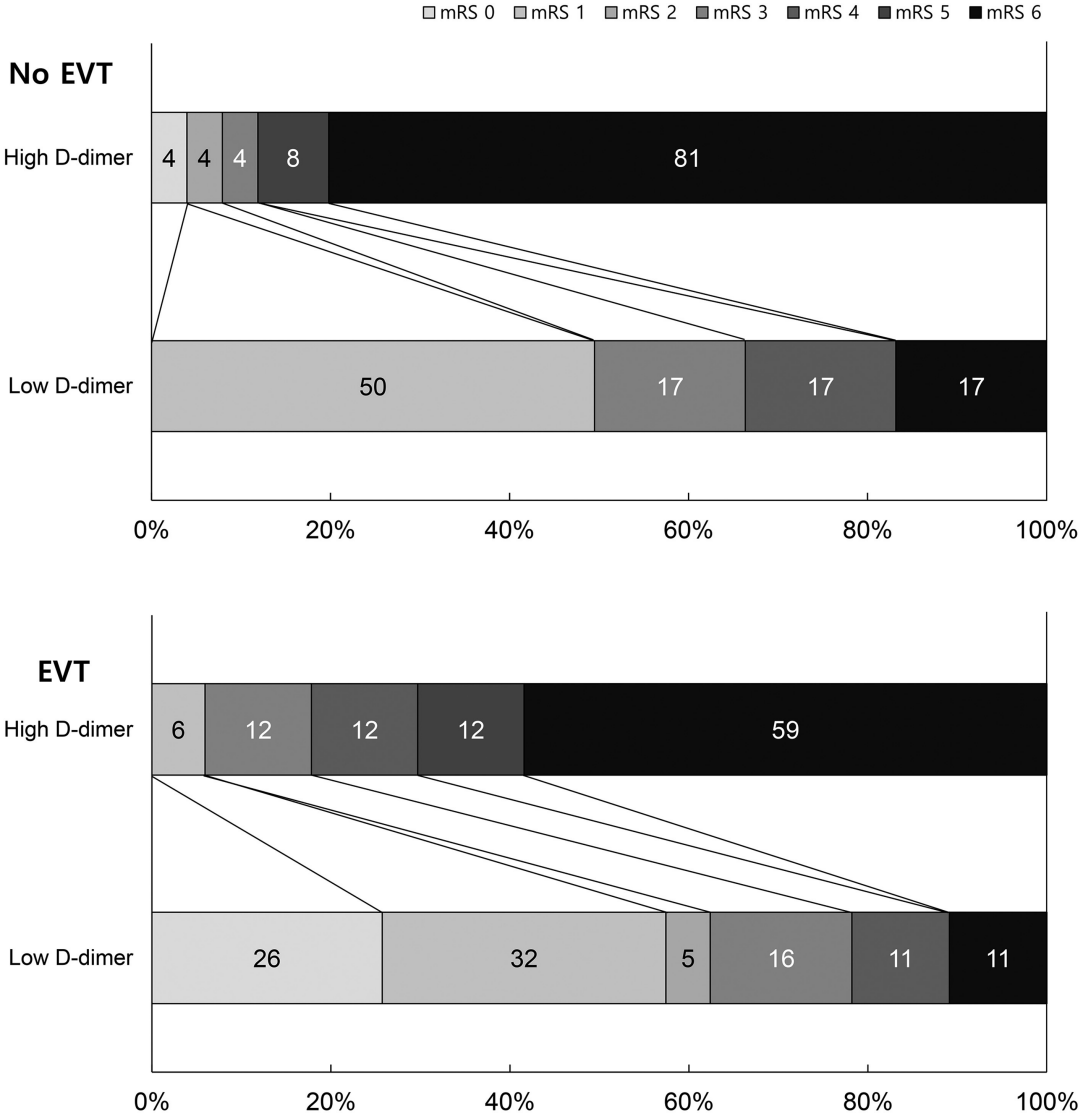
Distribution of modified 3-month Rankin Scale score by EVT and D-dimer group.

EVT and non-EVT groups showed poor 3-month mRS scores with high D-dimer levels. Patients with low D-dimer levels and EVT showed the best outcomes, while those with high D-dimer levels and non-EVT showed the worst outcomes (Figure 2). We also analyzed excluding the patients with identifiable other stroke etiologies such as atrial fibrillation to evaluate direct and exact influence of D-dimer on clinical outcomes. Supplementary Table 1 shows the Clinical outcomes by EVT and d-dimer groups excluding 11 atrial fibrillation patients. The results of clinical outcome by EVT and D-dimer groups were similar to previous data.

The cause of death was stroke-related in six patients (17.6%) and other medical problem–related deaths in 28 patients (82.4%). A total of 10 (27.8%) patients showed early dramatic neurologic improvement in the EVT group. Among them, 10 underwent EVT and reported early dramatic improvement, 3 (30%) died, and the other 7 (70%) were alive at 3 months. The seven survivors included six from the low D-dimer group and one from the high D-dimer group.

### Receiver Operating Characteristic Curve Analysis

**Figure 3:**
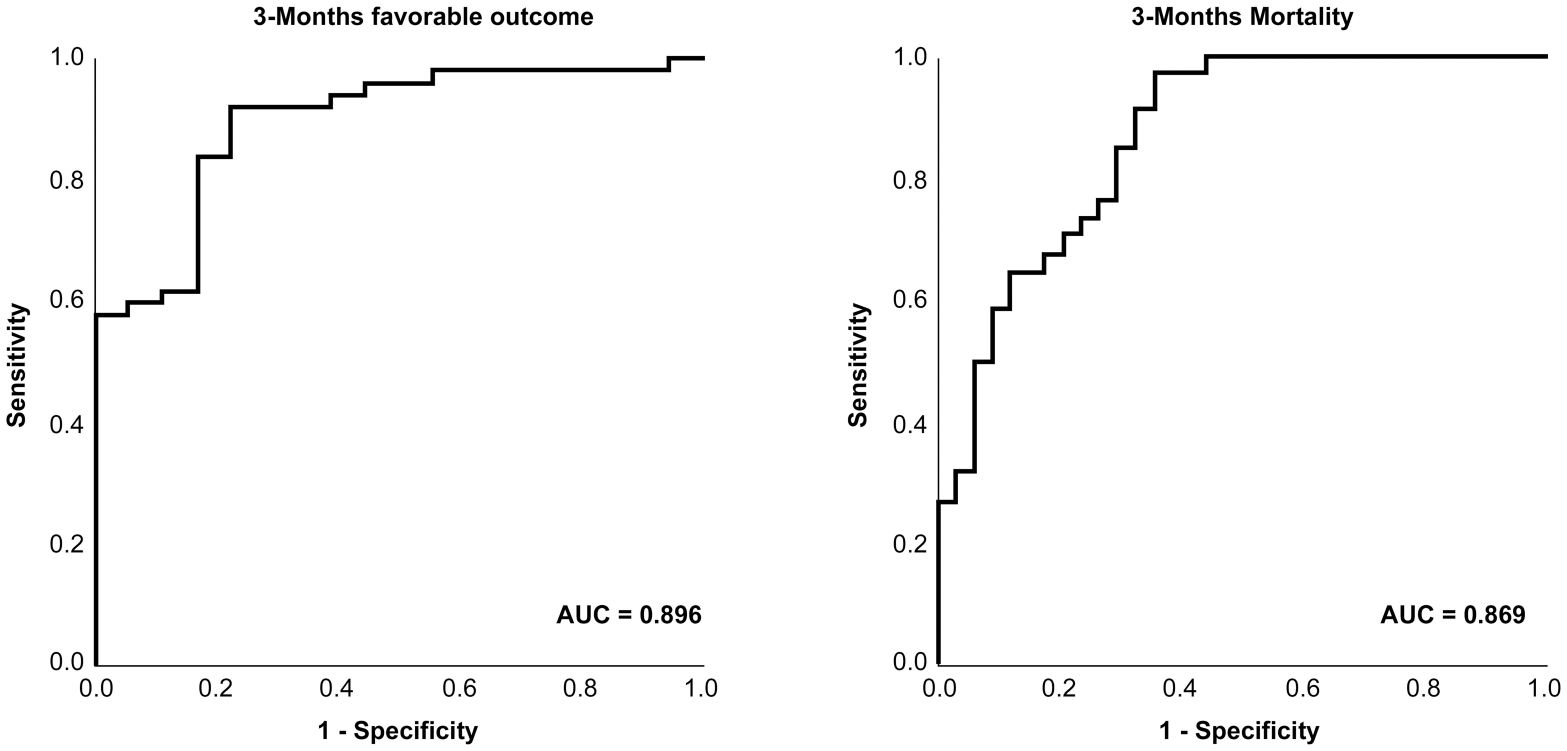
Receiver operating characteristic curve of D-dimer for the prediction of patient clinical outcomes.

Figure 3 shows the ROC of favorable outcomes and mortality by initial D-dimer level. The areas under the curve were 0.896(95% CI, 0.815–0.976) and 0.869(95% CI, 0.785–0.952), respectively. The initial D-dimer level was significantly related to a 3-month favorable outcome and mortality.

### Logistic Regression Analysis

Univariate and multivariate logistic regression analyses are presented in Table 3. The risk factors for 3-month mortality were EVT (OR, 0.297; *p* = 0.027) and an initially high D-dimer level (OR, 16.230; *p* < 0.001). The negative correlation factors of clinically favorable outcomes were lesion volume (OR, 0.936; *p* = 0.009) and high D-dimer levels (OR, 0.098; *p* = 0.005).

**Table 3:** Regression analysis of mortality and favorable outcomes.

	**Mortality**				**Favorable outcome**			
	**Univariate analysis**		**Multivariate analysis**		**Univariate analysis**		**Multivariate analysis**	
	**OR (95% CI)**	***P*-value**	**OR (95% CI)**	***P*-value**	**OR (95% CI)**	***P*-value**	**OR (95% CI)**	***P*-value**
Age	0.96 (0.91–1.01)	0.12			1.03 (0.97–1.09)	0.37		
Male sex	1.61 (0.62–4.19)	0.33			0.63 (0.21–1.86)	0.40		
Baseline NIHSS	1.05 (0.97–1.13)	0.25			0.94 (0.86–1.02)	0.14		
Hypertension	0.88 (0.33–2.36)	0.80			0.58 (0.18–1.87)	0.36		
Diabetes	1.29 (0.32–5.30)	0.72			1.47 (0.33–6.60)	0.62		
Hyperlipidemia	0.43 (0.12–1.61)	0.21			2.36 (0.64–8.71)	0.20		
Atrial fibrillation	0.51 (0.14–1.95)	0.33			1.05 (0.25–4.49)	0.95		
Smoking	0.88 (0.32–2.40)	0.80			0.68 (0.21–2.23)	0.53		
Alcohol	0.62 (0.16–2.44)	0.50			0.27 (0.03–2.28)	0.23		
Lung cancer	0.79 (0.30–2.05)	0.63			3.00 (0.97–9.30)	0.06		
Distant metastasis	2.05 (0.78–5.39)	0.15			1.15 (0.39–3.41)	0.80		
Hemoglobin	0.76 (0.60–0.97)	0.03	0.78 (0.60–1.01)	0.06	1.12 (0.88–1.43)	0.38		
Platelet count	0.99 (0.99–1.00)	0.03	1.00 (0.99–1.00)	0.20	1.01 (1.00–1.02)	0.01	1.01 (1.00–1.02)	0.03
LDL-cholesterol	1.00 (0.98–1.01)	0.68			1.01 (0.99–1.02)	0.51		
C-reactive protein	1.08 (1.00–1.17)	0.07			0.93 (0.83–1.03)	0.15		
**Occluded segment**								
MCA M1	1.45 (0.55–3.84)	0.46			0.49 (0.15–1.58)	0.23		
MCA M2	0.67 (0.25–1.85)	0.44			1.87 (0.62–5.66)	0.27		
Distal ICA	1.00 (0.34–2.94)	1.00			1.10 (0.33–3.67)	0.88		
Lesion volume, mL	1.01 (1.00–1.02)	0.10			0.93 (0.88– 0.98)	<0.01	0.94 (0.89– 0.98)	<0.01
Onset-to-ER	1.00 (1.00–1.01)	0.03	1.00 (1.00–1.01)	0.05	0.10 (0.99–1.00)	0.11		
Mechanical thrombectomy	0.23 (0.08–0.63)	<0.01	0.30 (0.10–0.87)	0.030	3.05 (0.95–9.85)	0.06	1.02 (0.19–5.37)	0.98
D-dimer >4	18.94 (4.78–75.16)	<0.01	16.23 (4.02–65.58)	<0.01	0.05 (0.01–0.21)	<0.01	0.10 (0.02–0.50)	0.05

## Discussion

A significant proportion of acute stroke patients with LVO had active cancer at presentation, a low successful recanalization rate, a higher probability of disability at 3 months, and higher mortality. D-dimer levels were independent prognostic factor for unfavorable outcomes and mortality among acute stroke patients with active cancer irrespective of EVT. In addition, D-dimer level shows an inversely correlated pattern depending on whether or not recanalization occurs. However, the sequential trend in each reperfusion grade was not statistically significant. Therefore, the treatment effect of EVT was affected by the baseline D-dimer level, suggesting that D-dimer can be a useful determinant of EVT in acute stroke patients with active cancer.

Previous studies showed a significantly worse prognosis in patients with active cancer than in those without cancer when treated with EVT (5, 6). These results suggest that conventional criteria for the decision of EVT were inappropriate for acute stroke patients with active cancer, suggesting the unmet need for new prognostic factors.

The etiology of stroke due to cancer-related coagulopathy is thought to differ from that of conventional stroke mechanisms. There have been emerging ideas of cancer-related stroke mechanisms, including intravascular coagulopathy, paradoxical embolisms with venous thromboembolism, and non-bacterial thrombotic endocarditis (12). Regarding these distinguishing etiologies of cancer-associated stroke, special consideration was made of unique stroke patterns involving multiple scattered lesions in multiple vascular territories that were associated with elevated D-dimer levels (3). D-dimer, a fibrin degradation product of a blood clot, is known to reflect coagulation mechanisms and hypercoagulability by direct measurement of activated coagulation and fibrinolysis (13). Several previous studies suggested the prognostic and diagnostic value of elevated D-dimer levels as a risk factor for stroke in patients with cancer or cardiovascular diseases (7, 8). Cancer patients with high D-dimer levels tend to show poor outcomes and a high mortality rate (14). Elevated D-dimer levels show the distinctive etiology of stroke but also reflect the general medical condition of patients with underlying cancer (8). Therefore, we hypothesized that the baseline D-dimer level in acute stroke patients with active cancer can be used to predict the overall outcome. Although the D-dimer level is used as a unique thrombosis biomarker, a specific level that predicts ischemic stroke is unknown. After the adjustment for EVT, our study showed that patients with elevated D-dimer levels above 4 μg/mL had about a 16-fold higher risk of death and a 90% lower chance of a favorable outcome at 90 days. Also in the EVT group, patients with elevated D-dimer levels above 4 μg/mL had about a 2.2-fold higher risk of death and a 90% lower chance of a favorable outcome at 90 days. We assumed that the D-dimer level might help guide whether to proceed with EVT and predict clinical outcomes in LVO cancer stroke patients.

However, considering the cause of mortality among our research results, the general medical condition is more important for prognosis than the stroke itself. Limited treatment EVT treatment effect in high D-dimer cancer patient is expected even with successful recanalization. In determining the EVT of cancer patients, in addition to the pre-existing criteria, general medical conditions such as D-dimer should be considered together. Approximately one-third of EVT cancer patients showed dramatic rapid neurological improvement, and the majority of had low initial D-dimer levels and mortality was numerically higher in the high D-dimer group (two-thirds of patients) despite the achievement of the early dramatic neurological improvement. Therefore, we should assess the initial D-dimer level when choosing EVT for a cancer patient, and if the D-dimer level is low, EVT can be considered appropriate.

Baseline D-dimer levels were associated with recanalization grade. The unique pathophysiology of intravascular coagulopathy, which often occurs in cancer patients, increases D-dimer levels (7). Tissue factors, inflammatory cytokines, or cancer procoagulants from cancer cells affect factor X activation in the coagulation cascade (3). This mechanism results in the formation of a solid fibrin clot within the vessel without any embolic thrombus. A previous study revealed that thrombi from EVT in cancer patients showed a significantly higher fraction of platelets and fibrin-rich components than those in non-cancer stroke patients (15, 16). D-dimer could be directly related to circulating tumor cells in cancer stroke patients as well (17). The characteristics of tumor embolism may be different from those of conventional RBC-rich clots or fibrin-rich clots. Further studies are needed to reveal the specific mechanism in cancer stroke patients.

This study had several limitations. First, a cautious approach is needed to interpret our results because this study was conducted in a single center and included a relatively small sample size. Due to small smaple size, the adjustment of recanalization rate of EVT was not possible and also other important variables such as baseline NIHSS, occlusion location were not included in multivariable analysis. Second, different cancer types and stages may have different effects on stroke. For example, disease progression rather than stroke itself might be a determinant of mortality at 3 months. Third, the etiologies of stroke in patients with cancer may be diverse and different etiologies could have contributed to stroke in this study. However, we confirmed that the main results of clinical outcomes were similar when the patients with atrial fibrillation were excluded.

Despite these limitations, our study revealed a significant relationship between initial D-dimer levels and clinical outcomes in patients with cancer-associated stroke and LVO. The initial D-dimer level might be a useful indicator to predict clinical outcomes and help clinicians decide whether to proceed with EVT.

## Conclusion

In conclusion, active cancer patients with LVO stroke treated with EVT showed low recanalization and poor clinical outcomes correlated with D-dimer levels. The initial D-dimer level was an independent factor predicting favorable clinical outcomes and mortality in patients with active cancer patients with LVO stroke. The results provisionally provide evidence that the decision of EVT should be cautiously made for cancer-associated stroke patients with LVO.

## Data Availability Statement

The raw data supporting the conclusions of this article will be made available by the authors, without undue reservation.

## Ethics Statement

The institutional review board of Samsung Medical Center approved the study protocol (2016-08-064). Because anonymized and open data were used in this study, the board waived the requirement for informed consent.

## Author Contributions

KP was produced study concept, interpreted data, and wrote the primary draft of manuscript. W-KS produced study concept, collected data, conducted statistical analysis, produced tables and figures, made a critical revision of the manuscript. The other authors collected and stratified patient data and made critical revision. All authors contributed to and approved the final manuscript.

## Funding

This study was funded by national research project ID 2020M3E5D2A01084891 from ministry of Health and Welfare in Republic of Korea.

## Conflict of Interest

The authors declare that the research was conducted in the absence of any commercial or financial relationships that could be construed as a potential conflict of interest.

## Publisher's Note

All claims expressed in this article are solely those of the authors and do not necessarily represent those of their affiliated organizations, or those of the publisher, the editors and the reviewers. Any product that may be evaluated in this article, or claim that may be made by its manufacturer, is not guaranteed or endorsed by the publisher.

## Abbreviations

EVT: endovascular thrombectomy
LVO: large vessel occlusion
mTICI: modified Treatment in Cerebral Infarction
mRS: modified Rankin Scale
NIHSS: National Institutes of Health Stroke Scale.
