# Supplementary material for: Significance of D-Dimer in Acute Ischemic Stroke Patients With Large Vessel Occlusion Accompanied by Active Cancer
Source: Front Neurol. 2022 Mar 23;13:843871. doi: 10.3389/fneur.2022.843871 (PMC8983900; doi:10.3389/fneur.2022.843871)
Supplement: Supplementary file 1 [file Table_1.docx]

**Supplemental Table 1.** Clinical outcomes by EVT and d-dimer groups excluding patients with atrial fibrillation

|  | Non-EVT  (n = 32) | EVT  (n = 25) | P value | d-dimer level < 4  (n = 17) | d-dimer level > 4  (n = 40) | P-value |
| --- | --- | --- | --- | --- | --- | --- |
| Successful recanalization (mTICI 2b–3) | - | 15 (60) | - | 9 (81.8)^*^ | 6 (42.9)^*^ | 0.04 |
| Hemorrhagic transformations | 3 (9.4) | 5 (20) | 0.54 | 0 (0) | 7 (17.5) | <0.01 |
| NIHSS at discharge | 16 (7 - 42) | 8 (2 - 14) | <0.01 | 5 (1 – 13) | 19 (16 – 42) | <0.01 |
| Median mRS score at 3 months | 6 (5 - 6) | 3 (1- 6) | <0.01 | 1 (1 – 3) | 5 (5 – 6) | <0.01 |
| Favorable outcomes (mRS score of 0–2) | 5 (15.6) | 10 (40) | 0.04 | 12 (70.6) | 3 (7.5) | <0.01 |
| Mortality at 3 months | 22 (68.8) | 8 (32) | <0.01 | 1 (5.9) | 29 (72.5) | <0.01 |

Values are number (%) or median (interquartile range).

* : Number of successful recanalization was calculated among EVT group only.
